# The *RET* gene encodes RET protein, which triggers intracellular signaling pathways for enteric neurogenesis, and *RET* mutation results in Hirschsprung's disease

**DOI:** 10.3934/Neuroscience.2022008

**Published:** 2022-03-16

**Authors:** Chacchu Bhattarai, Phanindra Prasad Poudel, Arnab Ghosh, Sneha Guruprasad Kalthur

**Affiliations:** 1 Department of Anatomy, Kasturba Medical College, Manipal, Manipal Academy of Higher Education, Karnataka, 576104, India; 2 Department of Anatomy, Manipal College of Medical Sciences, Pokhara, Nepal; 3 Department of Pathology, Manipal College of Medical Sciences, Pokhara, Nepal

**Keywords:** enteric neuron, gut wall, Hirschsprung's disease, neurogenesis, *RET* gene

## Abstract

Enteric neurons and ganglia are derived from vagal and sacral neural crest cells, which undergo migration from the neural tube to the gut wall. In the gut wall, they first undergo rostrocaudal migration followed by migration from the superficial to deep layers. After migration, they proliferate and differentiate into the enteric plexus. Expression of the Rearranged During Transfection (*RET*) gene and its protein RET plays a crucial role in the formation of enteric neurons. This review describes the molecular mechanism by which the *RET* gene and the RET protein influence the development of enteric neurons. Vagal neural crest cells give rise to enteric neurons and glia of the foregut and midgut while sacral neural crest cells give rise to neurons of the hindgut. Interaction of RET protein with its ligands (glial cell derived neurotrophic factor (GDNF), neurturin (NRTN), and artemin (ARTN)) and its co-receptors (GDNF receptor alpha proteins (GFRα1-4)) activates the Phosphoinositide-3-kinase-protein kinase B (PI3K-PKB/AKT), RAS mitogen-activated protein kinase (RAS/MAPK) and phospholipase Cγ (PLCγ) signaling pathways, which control the survival, migration, proliferation, differentiation, and maturation of the vagal and sacral neural crest cells into enteric neurons. Abnormalities of the *RET* gene result in Hirschsprung's disease.

## 1. Introduction

The enteric nervous system is part of the autonomic nervous system and is comprised of a complex array of interconnected neurons in ganglia located throughout the gut wall. The majority of neurons of the gut wall are derived from the vagal neural crest cells, with a minor contribution from the sacral neural crest cells [1]–[3]. These neural crest cells undergo massive migration, proliferation, and differentiation, an event that starts at about embryonic day 8.5 in animals and after the third week of intrauterine life in humans [4]. Several genes play an important role during the development of enteric neurons including the Rearranged During Transfection (*RET*) gene [5].

## 2. Origin and development of the enteric nervous system

The enteric nervous system is derived from the vagal and sacral neural crest of somite levels 1–7 and 28 [6]. These crest cells give rise to enteric neurons and ganglia of the pre-umbilical and post-umbilical parts of the gut wall [7]. They initially undergo a single wave of rostrocaudal migration along the gut wall [8],[9]. Only a small number of neural crest cells are required for rostrocaudal colonization in the gut wall [10],[11]. Next, they migrate to the unoccupied site of the developing gut and proliferate [12]. The migration of immature enteric neuroblasts in the gut wall takes place, on average, at a speed of 15 µm/h [13], which is slow compared to the undifferentiated vagal enteric neural crest cells [13],[14]. A second wave of migration occurs from the periphery to the deep layers of the gut wall through connective tissue pathways, forming the myenteric plexus first, followed by the submucosal plexus [15]. In birds, the enteric neural crest cells first migrate toward the submucosa, forming the submucosal plexus, which then migrates outwards between muscle layers to form the myenteric plexus [16]. During the morphogenesis and differentiation of enteric neural crest cells into enteric neurons, several proteins [17],[18] play essential roles. For the differentiation of vagal and sacral neural crest cells into enteric neurons, the *RET* gene and the encoded RET protein play a pivotal role [19],[20].

The RET protein is a receptor tyrosine kinase. The *RET* gene was originally described as a human oncogene, but it was later established that RET plays a crucial role in the development of enteric neurons and defects in the human *RET* gene result in the syndrome known as Hirschsprung's disease [21].

## 3. Location and structure of the *RET* gene and protein

### 3.1. Location of the *RET* gene

The *RET* gene lies in the long arm of chromosome 10 (10q11.2) and contains 21 exons [22] and 18 or 5 introns [23],[24]. The DNA sequence of this gene was originally found to be rearranged within 3T3 fibroblast cell line following transfection with DNA from lymphoma cells [25].

### 3.2. Structure of the RET protein

The *RET* gene encodes a receptor tyrosine kinase transmembrane protein [26]. The RET protein has three different isoforms (RET51, RET43, and RET9), which differ in the C-terminal amino acids [27]. Two isoforms, RET9 and RET51, also differ in their intracellular domains [28],[29]. RET is comprised of 1114 amino acids [30] and has three domains. The N-terminal domain is extracellular and consists of 29–635 amino acids [30]. It has four cadherin-like domains (CLDs) and cysteine-rich regions [31]–[33]. The CLDs each consist of 110 amino acids [34] and CLD2 and CLD3 each have a Ca^2+^ binding site, which is required for maintaining the integrity of the RET protein [35]. The cysteine-rich regions contain 120 residues and are connected to the transmembrane domain [34]. The hydrophobic transmembrane domain of RET spans the cell membrane [31],[33] and consists of 636–657 amino acids [30]. It mediates extracellular calcium-binding for maturation of the immature 150-kDa RET protein in the endoplasmic reticulum to the mature 170-kDa protein and its migration to the cell membrane [36],[37]. Finally, the cytoplasmic domain is a tyrosine (Tyr) kinase domain and consists of 657–1114 amino acids [30]. It contains 16 tyrosine residues (six in RET9, 18 in RET51, whereas Tyr 1090 and Tyr 1096 are present only in RET51) [31],[32]. This domain also contains catalytic protein kinases, a distinct regulatory sequence of 14–18 tyrosine residues, and serine and threonine phosphorylation sites [38],[39]. There are 18 tyrosine residues, two in the juxtamembrane domain, 11 in the kinase domain, and five in the carboxyl terminal tail [40]. In addition, this domain also has phosphopeptide motifs that provide a binding and docking site for cytoplasmic downstream signaling proteins, such as Src homolog 2 (SH2) and phosphotyrosine-binding domain (PTB) [41].

### 4. Intracellular signaling pathways of RET for enteric neurogenesis

**Figure 1.**
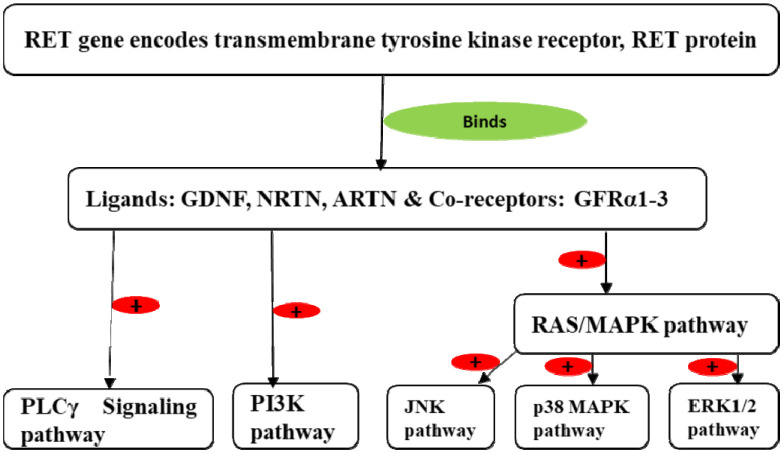
Schematic representation of intracellular signaling pathways of RET.

The RET protein is a member of the glial cell-derived neurotrophic factor (GDNF) family of extracellular signaling molecules [25],[42]. The RET ligand GDNF [1],[43] is a dimeric growth factor protein related to a member of the transforming growth factor-beta (TGF-β) superfamily. This superfamily has four additional subtypes: GDNF, neurturin (NRTN) [44], persephin (PSPN) [45], and artemin (ARTN) [46]. They bind with the RET protein via its co-receptors, the GDNF receptor alpha proteins (glycosylphosphatidylinositol (GPI) anchored co-receptor family) GFRα1, GFRα2, GFRα3, and GFRα4 [47]–[49]. The cysteine-rich extracellular CLD4 domain of RET makes a direct crosslink with GFRα1 [50] and the CLD1–3 domains [51] fold into a compact shell [52]. This maintains the conformation of RET during binding [50]. The extracellular domains form ternary complexes of their ligand, co-receptor, and the receptor RET protein as follows: *i*) GDNF with GFRα1 and RET; *ii*) NRTN with GFRα2 and RET; *iii*) ARTN with GFRα3 and RET. These ternary complexes induce dimerization of the RET protein. During dimerization, there is trans-autophosphorylation of Tyr905 and Tyr900 of the tyrosine kinase domain of the RET protein, which further autophosphorylates other tyrosine residues (Tyr981, Tyr1015, Tyr1062, Tyr1063, and Tyr1096) [39],[53]. Phosphorylation of Tyr1096 takes place only in the RET51 isoform. Phosphorylation of Tyr1062 of the tyrosine kinase domain of the RET protein activates RAS/MAPK and PI3K-PKB/AKT pathways [40],[41],[54],[55] while autophosphorylation of other tyrosine residues induces PLC-γ and JNK pathways [47] (Figure 1).

#### 4.1. RAS/MAPK pathway

**Figure 2.**
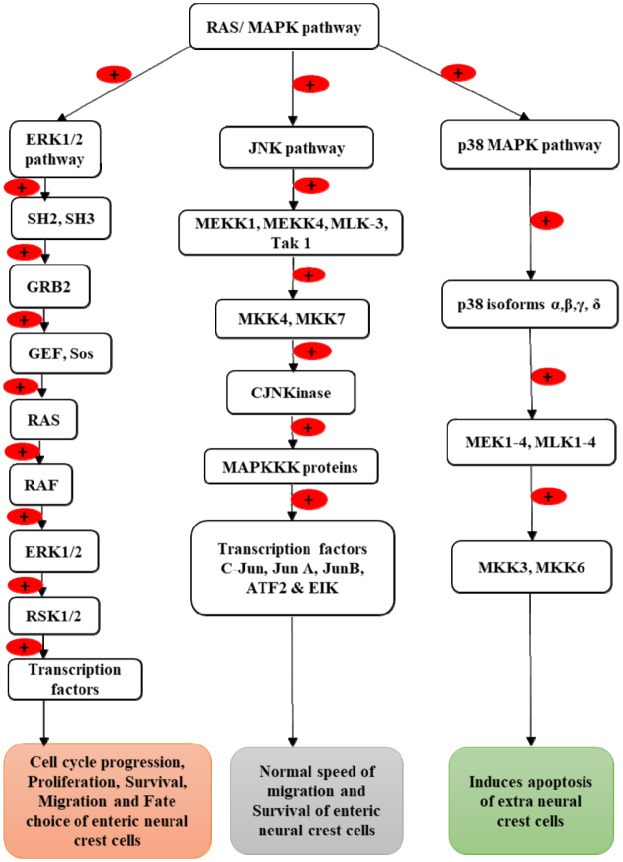
RAS/MAPK pathway.

Phosphorylation of serine, threonine, and tyrosine of the activation loop of the tyrosine kinase domain of the RET protein stimulates mitogen-activated protein kinase kinases (MAPKK), which are upstream of the MEK proteins. MEKs are activated by various upstream activators, including kinases and small GTP binding proteins. MEK then activates three MAPK [56] pathways: extracellular signal-regulated kinase 1/2 (ERK1/2), JNK, and p38 mitogen-activated protein kinase (p38 MAPK) (Figure 2). All three pathways consist of three-tiered kinase cascades that phosphorylate hundreds of substrates in the cytoplasm and nucleus, leading to cellular proliferation, survival, apoptosis, migration, and differentiation [57].

##### 4.1.1. ERK1/2 pathway

Upon activation, the RET protein tyrosine kinase domain [58] binds to Src homolog 2 and 3 (SH2 and SH3) domains of phosphotyrosine. SH2 and SH3 bind to adaptor molecule GRB2 [59],[60], which interacts with the guanine nucleotide exchange factor (GEF) Sos (Son of sevenless) [61]. Sos then promotes the exchange of GDP for GTP on the RAS protein, which binds and activates the MAPKK kinase protein, RAF. RAF then phosphorylates threonine and tyrosine residues on the activation loop of the MAPK protein, ERK1/2, which further phosphorylates multiple cytoplasmic and cytoskeletal proteins [56],[62] such as MAPK-activated protein kinases and ribosomal S6 kinases (RSK). ERK and ribosomal S6 kinases 1/2 (RSK1/2) then translocate into the nucleus. ERK phosphorylates and activates several transcription factors, including SP, E2F, ELK-1, AP-1 [63], ELK-7, FOS, Myc, and MEF2 [62],[64],[65]. RSK1/2 activates big MAP kinase (BMK1), i.e., ERK5 [62],[66], and it phosphorylates several transcription factors including Myc, MEF2 family members, FOS, and serum- and glucocorticoid-inducible kinase (SGK). Together, these transcription factors lead to cell cycle progression [67], proliferation, survival, migration, and fate choice of cells [68],[69].

##### 4.1.2. Jun-mediated signaling pathway

The JNK pathway is required for the normal migration of enteric neural crest cells. Several MAPKKKs together with MEKK1–4, MLK3, and Tak1 phosphorylate and activate MKK4 and MKK7 [70]. MKK4 and MKK7 then catalyze the phosphorylation of C-Jun N-terminal kinase (CJN Kinase) [71]. This further activates MAPKKK via the small G-protein, RAC. RAC further activates MLK3, MEKK1, and MEKK4 [72]–[74], and finally activates the JNK pathway. This JNK pathway then causes the phosphorylation and activation of several transcription factors, including C-Jun, Jun A, Jun B, ATF2, and EIK, and these enable enteric neural crest cell survival [70] and migration [75],[76].

##### 4.1.3. p38 MAPK pathway

Upon phosphorylation of the tyrosine kinase domain of the RET protein, it activates the four p38 isoforms, α, β, γ, and δ [77]. These p38 isoforms activate several MAPKKKs, including MEK1–4 and MLK1–4, which further activate MKK3 and MKK6, and thus induce apoptosis of extraneural crest cells [71].

#### 4.2. PLCγ signaling pathway

**Figure 3.**
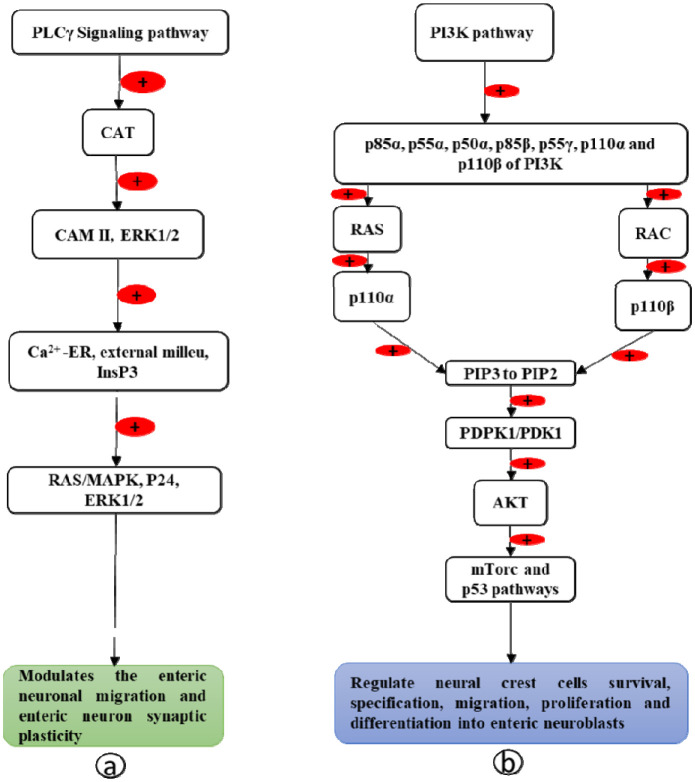
a. PLCγ signaling pathway and b. PI3K pathway.

PLCγ contains two SH2 domains and one SH3 domain. The SH2 domains bind phosphotyrosine and the SH3 domain binds the proline-rich sequences of RET [78],[79]. Upon ligand (GDNF) stimulation, there is phosphorylation of Tyr1015 and Tyr1016 of the tyrosine kinase domain of the RET protein. Through the PLCγ binding domain [80], the RET protein recruits the transmembrane adaptor, CAT protein [81]. CAT activates calcium calmodulin-dependent kinase II (CAMK II) and ERK1/2 [82],[83], which causes the release of Ca^2+^ from the endoplasmic reticulum and extracellular milieu [84],[85] through the inositol 1,4,5-triphosphate (InsP3) receptor (InsPR). The released Ca^2+^ then triggers RAS/MAPK by phosphorylating p42/44 of MAPK (ERK1/2). This modulates the enteric neuronal migration and enteric neuron synaptic plasticity [85],[86] (Figure 3. a).

#### 4.3. PI3K pathway

Upon stimulation by its ligand (GDNF), the tyrosine kinase domain of the RET protein binds with regulatory subunits p85α, p55α, p50α, p85β, p55γ, p110α, and p110β of PI3K enzymes via its phosphotyrosine-binding SH2 domain [87]. Then, the catalytic subunits p110α (activated by G-protein RAS) and p110β (activated by G-protein RAC) [88] degrade the phosphatidylinositol (3,4,5)-trisphosphate (PIP3) to phosphatidylinositol (4,5)-bisphosphate (PIP2) by phosphatase [89]. This PIP2 activates 3-phosphoinositide-dependent protein kinase-1 (PDPK1/PDK1) [90] and phosphorylates AKT [91],[92]. The activated AKT then regulates neural crest cell survival, specification, migration, proliferation, and differentiation into enteric neuroblasts [93] via the mTorc and P53 pathways [94] (Figure 3. b).

### 5. Abnormalities of the *RET* gene

Deletion of the *RET* gene or mutations in the exons and introns that result in changes in the intracellular and extracellular domains of RET lead to Hirschsprung's disease (Tables 1–11).

**Table 1.** Germline mutations of the *RET* gene.

Mutations	Domains affected	Exons affected	Introns affected	Codons affected	Outcomes
Germline mutation [95],[96]	Extracellular domain [95],[96]	10	-	609, 611, 618, 620	Hirschsprung's disease
		11	-	630, 634	
	Intracellular domain-tyrosine kinase 1 residue of tyrosine kinase domain [95],[96]	15	-	883, 891	
		14	-	804	
		15	-	883, 891, 918	
Germline mutation [97]	-	10	-	c.1852 T>C	

**Table 2.** Nonsense, frameshift, and point mutations of the *RET* gene.

Mutations	Domains Affected	Exons affected	Introns affected	Codons affected	Outcomes
Nonsense mutation	Extracellular domain [98]	2, 3, 4, 5, 6 [98]	-	-	Hirschsprung's disease
	Amino acid substitution [98]–[101]	-	-	-	Familial or sporadic cases of Hirschsprung's disease
Frameshift mutation	*RET* gene	-	-	Phe147del [102]	Hirschsprung's disease
	Amino acid substitution in protein truncation of RET [98]–[101]	-	-	-	Familial or sporadic cases of Hirschsprung's disease
Point mutation	*RET* gene in heterozygous state [24],[98],[103]	-	-	-	Hirschsprung's disease

**Table 3.** Missense mutations of the *RET* gene.

Mutations	Domains Affected	Exons affected	Introns affected	Codons affected	Outcomes
Missense mutation	Extracellular domain [98]	2, 3, 4, 5, 6 [98]	-	-	Hirschsprung's disease
	Impair the RET kinase activity leading to the impairment of the phospholipase C-γ signaling pathway [104]	-	-	E762Q, S767R, R972G, M900T [104]	
	Complete loss of RET kinase activity [104]	-	-	S765P, R873Q, F893L, R897Q, E921K [104]	
	RET tyrosine kinase domain [23]	-	-	-	
	Dominant negative effect through loss of function [24],[105],[106]	-	-	-	
	-	15 [107]	-	At nucleotide 2813G to A with R873Q exchange in codon 873 [107]	
		3 [108]	-	Nucleotide change GTG to ATG (V202M mutation) [108]	
		7 [108]	-	Nucleotide change GAA to AAA (E480K mutation) [108]	Rectosigmoidal aganglionosis
		17 [108]	-	Nucleotide change CCA to ATA (P973L mutation) [108]	
		13 [108]	-	Nucleotide change GAC to AAC (D77/N mutation) [108]	Total gut wall aganglionosis
	Amino acid substitution in RET protein [98]–[101]	-	-	-	Familial or sporadic cases of Hirschsprung's disease

**Table 4.** Deletions in the *RET* gene.

Mutations	Locations	Outcomes
Deletion [103]	*RET* gene [103]	Hirschsprung's disease In 20% patient have low efficiency in detection of deletion [103]
Partial deletion [109]	*RET* locus at pericentromeric region of chromosome 10 [109]	
Interstitial deletion [110]	In the long arm of chromosome 10- del10(q11.21, q21.2) [110]	Total colonic aganglionosis and minor involvement of myenteric plexus [110]
Proximal deletion [111]	In the long arm 10- del10q11.2 to q21.2 Deletion location likely lying between loci D10S208 and D10S196 [111]	Colonic aganglionosis in hindgut [111].
Cytogenetic deletion [112]	del (10) (q11.2 to q21.2) [112]	Total aganglionosis with small bowel involvement [112]

**Table 5.** Mutations of the extracellular domain of the RET protein.

Mutations	Locations	Effects	Outcomes
Mutation in extracellular domain	N terminus region of RET protein [113]–[115]	Affect the amino acid residue No glycosylation of immature 150-kDa form in the endoplasmic reticulum No production of mature 170k-Da form of RET protein No expression of RET protein in cell membrane [113],[114]	Hirschsprung's disease [113]–[115]

**Table 6.** Mutation of intracellular domain of the RET protein.

Mutations	Locations	Effects	Outcomes
Mutation in intracellular domain	Tyrosine kinase domain [23]	Impaired intracellular signaling pathways [23]	Hirschsprung's disease
	Tyrosine kinase domain 1 [Glu 762-Gln (E762Q), Ser65 to Pro (S765P) and Ser767 to Arg (S767R)] or tyrosine kinase domain 2 [Arg 873 to Gln (R897Q), Glu 921 to Lys (E921K), Arg 972 to Gly (R972G), Pro 973 to Leu (P973L) and Met 980 to Thr (M980T)] [24],[98]–[100],[116]–[119]	-	Familial and sporadic Hirschsprung's disease [24],[98]–[100],[116]–[119]
	Tyrosine residue at position 1062 which is intracytoplasmic docking site of RET protein [120]	Impaired fixation of SHc to RET protein and thus prevention of the phosphorylation and inhibition of the signaling pathway and thus exert negative effect in the enteric neurogenesis [120]	Hirschsprung's disease

**Table 7.** Insufficient expression of the *RET* gene.

Expression of *RET* gene	Effects	Outcomes
Insufficient level of expression	Insufficient expression of RET protein on the cell surface for GDNF and its co-receptor GFR α1–4 [101],[121]	Hirschsprung's disease

**Table 8.** Mutations in exons of the *RET* gene.

Mutations	Locations	Effects	Outcomes
Mutation in exons	Exon 2 in codon 32 changing CTG to TTG [98]	Changes the protein sequence of extracellular domain of RET from serine to leucine [98]	Congenital absence of enteric neurons and ganglia in intestine [98]
	Exon 3 in codon 180 changing CGA to TGA [98]	Changes the protein sequence of extracellular domain of RET from arginine to stop codon [98].	
	Exon 5 in codon 330 changing CGG to CAG [98]	Changes the protein sequence in extracellular domain of RET from arginine to glutamine [98]	
	Exon 6 in codon 393 changing TTC to TTA [98]	Changes the protein sequence of extracellular domain of RET from phenylalanine to leucine [98]	
	Exon 10 with nucleotide change of C1876A and amino acid change of Q6226K [122]	-	Sporadic ultra-short-segment aganglionosis [122]
	Exon 11 with nucleotide change of C1941T and amino acid change of 16471 [122]	-	Sporadic long-segment aganglionosis [122]
	Exon 10 with change in five cysteine codons from Cys to Trp at codon 699 and Cys to Arg at codon 618 or 620 [97]	-	Hirschsprung's disease
	Exon 2 with change in nucleotide from C254 G to A [123]	Loss of function of RET gene [123]	Total colonic aganglionosis [123]
	Exon 13 with change in nucleotide from C2308 C to T [123]		
	Exon 14 with change in nucleotide from C2578 C to T [123]		
	Exon 4 with change in nucleotide from C789 C to G [123]	-	Long segment Hirschsprung's [123]

**Table 9.** Mutations in the *RET* gene affecting RET protein isoforms.

Mutations	Effects	Outcomes
RET isoforms mutations and defects	RET9 [124]	Lack of enteric ganglion in colon [124]
	Mutation of tyrosine 1062 of RET9 to phenylalanine [125]	Deficient in enteric nervous system [125]

**Table 10.** Mutations in enhancer, promoter, and introns of the *RET* gene.

Mutations	Locations	Effects	Outcomes
Mutation of enhancer [126]	Enhancer domain of the *RET* gene in intron 1 (CrS2435357) [126]	-	Hirschsprung's disease with significantly higher impact in males than females [126]
Promoter defect [127]	Methylation of promoter of *RET* has 5′ CC-3′ [127]	-	Colonic aganglionosis [127]
Mutation in introns	Alteration in intron 4 at putative branch site of 24 nucleotides in front of exon 15 with nucleotide exchange of G to A [107]	-	Hirschsprung's disease [107]
	Missense mutation in intron 19 (IVS 19-19 C/T) [108]	-	Only rectosigmoidal aganglionosis [108]

**Table 11.** Homozygous and heterozygous mutations of the *RET* gene.

Mutations	Locations	Effects	Outcomes
Homozygous *RET* mutations	C620R mutation [128]	-	Hirschsprung's diseases [128]
	Mutation of tyrosine 1062 in RET with phenylalanine [129]	Impairing the binding site of tyrosine 1062 for phosphotyrosine-binding domains for several adaptors and effector proteins which otherwise are important for activation of intracellular signaling pathways, such as RAS/ERK, phosphatidylinositol 3-kinase/AKT, and Jun-associated N-terminal kinase pathways [129]	Severe defect in the development of enteric nervous system in 40% of cases [129]
	Homozygous missense mutation (CGG to TGG) at codon 969 of RET with amino acid change from arginine to tryptophan [130]	Critical alteration in RET tyrosine kinase activity [130]	Total gastrointestinal tract aganglionosis [130]
Heterozygous *RET* mutation [128]	C620R mutation [128]	-	Hirschsprung's disease including hypoganglionosis of gastrointestinal tract [128]

### 6. Conclusions

Vagal and sacral neural crest cells migrate in a rostrocaudal direction where they colonize in an orderly manner in the foregut, midgut, and hindgut following signaling by the receptor tyrosine kinase RET protein. This protein promotes the survival of enteric neurons, as well as proliferation and differentiation of multipotent enteric progenitor cells present in the gut wall. Developmental studies in model organisms and genetic studies of Hirschsprung's disease have provided a detailed understanding of enteric nervous system development via expression of the *RET* gene. In summary, the *RET* gene encodes a tyrosine kinase receptor, RET, which is required for the normal formation of enteric neurons. Mutation of the *RET* gene leads to dysfunctional RET binding to the GDNF, ARTN, and NRTN ligands resulting in Hirschsprung's disease.
